# Causal Model Building in the Context of Cardiac Rehabilitation: A Systematic Review

**DOI:** 10.3390/ijerph20043182

**Published:** 2023-02-11

**Authors:** Nilufar Akbari, Georg Heinze, Geraldine Rauch, Ben Sander, Heiko Becher, Daniela Dunkler

**Affiliations:** 1 Institute of Biometry and Clinical Epidemiology, Charité—Universitätsmedizin Berlin, Corporate Member of Freie Universität Berlin and Humboldt-Universität zu Berlin, Charitéplatz 1, 10117 Berlin, Germany; 2 Center for Medical Data Science, Institute of Clinical Biometrics, Medical University of Vienna, Spitalgasse 23, 1090 Vienna, Austria; 3 Technische Universität Berlin, Straße des 17, Juni 135, 10623 Berlin, Germany; 4 Institute of Global Health, University Hospital Heidelberg, Im Neuenheimer Feld 130.3, 69120 Heidelberg, Germany

**Keywords:** causal regression modeling, variable selection, functional forms, cardiac rehabilitation

## Abstract

Randomization is an effective design option to prevent bias from confounding in the evaluation of the causal effect of interventions on outcomes. However, in some cases, randomization is not possible, making subsequent adjustment for confounders essential to obtain valid results. Several methods exist to adjust for confounding, with multivariable modeling being among the most widely used. The main challenge is to determine which variables should be included in the causal model and to specify appropriate functional relations for continuous variables in the model. While the statistical literature gives a variety of recommendations on how to build multivariable regression models in practice, this guidance is often unknown to applied researchers. We set out to investigate the current practice of explanatory regression modeling to control confounding in the field of cardiac rehabilitation, for which mainly non-randomized observational studies are available. In particular, we conducted a systematic methods review to identify and compare statistical methodology with respect to statistical model building in the context of the existing recent systematic review CROS-II, which evaluated the prognostic effect of cardiac rehabilitation. CROS-II identified 28 observational studies, which were published between 2004 and 2018. Our methods review revealed that 24 (86%) of the included studies used methods to adjust for confounding. Of these, 11 (46%) mentioned how the variables were selected and two studies (8%) considered functional forms for continuous variables. The use of background knowledge for variable selection was barely reported and data-driven variable selection methods were applied frequently. We conclude that in the majority of studies, the methods used to develop models to investigate the effect of cardiac rehabilitation on outcomes do not meet common criteria for appropriate statistical model building and that reporting often lacks precision.

## 1. Introduction

Cardiac rehabilitation (CR) is a secondary cardiovascular prevention strategy consisting of programs that include for example physical exercise, health education and stress management [1]. The programs are supervised interventions for patients with heart diseases intending to reduce cardiovascular risk and improve the prognosis of survivors and lifestyle management [1]. Table 1 summarizes the main components of CR programs according to Rauch et al., 2016 [2], Rauch et al., 2014 [3] and Dalal et al., 2015 [1]. CR programs are not standardized and may contain subsets of these components. They vary widely in many aspects between and within countries and thus exhibit a high degree of heterogeneity. Very precise inclusion and exclusion criteria are necessary for CR programs to be comparable [4]. Randomized clinical trials are rare in this context since participation in CR programs is supported by different facilities such as government policy, health insurance and pension funds in most countries. Ethical and practical considerations may further complicate randomization. Hence, mainly observational studies have been conducted to evaluate the effect of CR programs, making it difficult to generalize findings [2].

Generally, the effect of an intervention on patient outcomes can optimally be assessed when treatment assignments are randomized such that the baseline characteristics of patients in the different treatment groups are, on average, structurally balanced. Randomized controlled trials ensure that differences in outcomes can only be attributed to the type of intervention and reduce the likelihood that structural differences between groups affect the effect estimates.

In non-randomized studies, a statistical model may help in making the intervention groups comparable by mathematically equalizing structural differences. However, statistical model building for the model-based adjustment of the causal effect of a non-randomized intervention in observational studies is a complex task. Both statistical expertise and subject matter knowledge are essential to build an appropriate statistical model that allows the identification of the effect of the intervention. In particular, the assumptions about causal relationships between potential confounding variables, the intervention and the outcome can be portrayed in a directed acyclic graph (DAG), and based on this DAG, an appropriate set of confounders to include as adjustment variables in a statistical model can be identified [5]. By contrast, data-driven variable selection does not support this process and should be avoided for that purpose [6,7,8]. While DAGs may help in identifying confounders to adjust for, they do not make any assumptions on whether the association of the outcome with continuous confounders should be assumed to be linear, log-linear, or even more complex such as U-shaped or S-shaped. Hence, besides the selection of variables for models, the specification of the functional form of their association with the outcome is an essential part of model building, and it is difficult to balance strong assumptions (e.g., linearity) that may lead to poor model fit against an exaggerated flexibility of a model that may finally lead to overfitting the data [9]. Further methodological challenges are the handling of missing values [10] and ensuring model robustness [11].

While the statistical literature gives a variety of recommendations on how to build multivariable regression models in practice [9,12,13], our hypothesis is that this statistical guidance is often unknown. Hence, we set out to describe the current practice of model building and to identify any gaps between what has been proposed from a methodological point of view, and what is actually used in practice in a setting where non-randomized studies are common. The updated Cardiac Rehabilitation Outcome Study (CROS-II) by Salzwedel et al. [14] was a systematic review of studies to evaluate the effect of CR. We extended this systematic review by evaluating the statistical methodology for causal model building used in the included non-randomized interventional studies. We also investigated whether the methods were reported in sufficient detail so that studies could be successfully replicated and the methodological quality could be assessed. A secondary goal of our systematic methods review was to identify which variables were considered as confounders in the reported models of the screened studies.

## 2. Methods

This systematic review was not preregistered. The structure follows the PRISMA guidelines and a checklist addressing the items can be found in the supplementary materials (Supplementary S1 Checklist). A review protocol was not prepared.

### 2.1. Sources of Information and Search Strategy

CROS was a multicenter review and meta-analysis project that was first published with 25 studies in 2016 [2] and updated (CROS-II) with six additional studies in 2018 [14]. The study also addressed the fact that the types of CR programs vary widely across and within countries on many issues, and that there are no accepted minimum standards for assessing the quality of CR programs worldwide. The inclusion criteria of CROS had restrictions regarding cardiac rehabilitation programs, whereby start, supervision, so-called ”multi-component” CR programs, and CR setting were defined. Multi-component was defined as ”CR including supervised and structured physical exercise at least twice a week as basic requirement plus at least one, preferably more, of the following components: information, motivational techniques, education, psychological support and interventions, social and vocational support” ([2]; p. 1915). The control group in all the studies consisted of patients receiving standard care. In addition, a restriction was also raised with regard to the statistical methods requiring that the cohort studies must have had a description of the data sources and should also have used methods to reduce risk and selection bias such as regression modeling. Altogether, in CROSS-II 25, 630 titles were screened. All studies published until 4th September 2018 meeting the eligibility criteria were included. In total, 31 studies, with total mortality (all-cause mortality) as the primary outcome and cardiac rehabilitation as the intervention, were included in CROS-II.

### 2.2. Selection of Studies for the Systematic Methods Review

**Figure 1:**
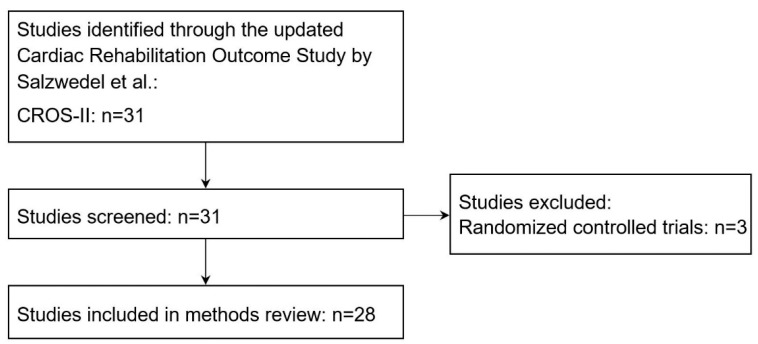
Flow diagram of selection of studies.

Figure 1 provides an overview of the selection of studies for this review. Of the 31 studies that were selected for CROS-II, three were excluded for this systematic methods review because the studies were randomized controlled trials [15,16,17] and not observational studies. Two of these RCTs were standard randomized clinical trials; the patients were randomized into a standard/usual care group and an exercise-based rehabilitation group. However, the sample size in both studies was comparably small with 36 [16] and 204 participants [17] compared to a median sample size of 1474 (IQR: 677; 3560) in the other studies included in CROS-II. In the third manuscript [15], the authors reported two studies: The first one was a standard RCT where 1813 participants were randomized into CR programs or usual care after hospital discharge. In the second pragmatic study, 331 patients from four different hospitals were included. Participating hospitals that already referred most of their eligible patients for CR (elective rehabilitation hospitals) were matched to hospitals where this was not the case (elective control hospitals). Subsequently, all eligible and consenting patients from elective rehabilitation hospitals were referred for CR, while all eligible and consenting patients from elective control hospitals were put in the control group. The remaining 28 studies served as basis of this paper.

### 2.3. Data Management, Collection Process and Data Items

For the purpose of data extraction from the 28 included studies, we designed and used a content extraction sheet (Supplementary S1 File) collecting information on study and modeling characteristics. Extracted data was collected in an electronic database.

Data was collected independently from the studies by two raters (NA + BS) and discrepancies were resolved by subsequent discussion. In addition to the methodological description in the articles, we also used any published supplementary materials and consulted the documentation of the software used, such as R packages, to identify the applied methods.

Extracted meta-data were:

**Study characteristics** including study design, name of first author, publication year and sample size.

**Modeling characteristics** including variable selection procedures, functional form of continuous variables, use of propensity scores, general aspects of regression modeling, type of regression model, and selected covariates. In total, 36 aspects of regression modeling were examined.

### 2.4. Summary Measures and Risk of Bias Assessment

A quality assessment of the studies was already performed in CROS-II and was summarized in two tables, one for observational studies ([14], Table 3) and one for RCTs ([14], Table 4). To assess quality in the observational studies, the checklists of methodological issues on non-randomized studies [18,19] and the Newcastle Ottawa Scale (NOS) were used. We extended the table for observational studies from CROS-II ([14], Table 3) to evaluate aspects of statistical analysis and model building with a causal aim, according to the criteria listed in Table 2. These additional aspects are based on the guidance documents published by the international ‘Strengthening Analytical Thinking for Observational Studies’ (STRATOS) initiative (https://www.stratos-initiative.org: accessed on 9 January 2023) and additional sources [20].

Table 2 shortly summarizes some aspects of statistical analysis, including initial data analysis, variable selection and assumption about functional forms, which should be taken into consideration when analyzing an observational study, and we provide appropriate references for guidance. These aspects were evaluated in the CR studies selected for this systematic methods review using an assessment procedure as described in the right column of Table 2. To evaluate the variable selection methods in regression analyses, the studies were classified according to the categories given in Table 3. We distinguished whether the variables were selected for building the propensity score or directly for inclusion as confounders in outcome models. The identified methods were reported in the results section in the same way as they were described in the original papers.

## 3. Results

Of the 28 observational studies, four studies did not adjust for confounders [30,31,32,33]. Therefore, the following results refer only to the 24 studies that used specific methods to adjust for confounding. The study characteristics are listed in the supplementary materials (Supplementary S2 File).

In total, there were eleven studies (46%), which did not report how they came up with the selected variables [3,34,35,36,37,38,39,40,41,42,43,44]. Four studies (17%) employed only univariable screening and included the significant variables from the univariable model in the final model [39,45,46,47]. Two studies (8%) included variables only on the basis of background knowledge [48,49]. One study (4%) determined the final model with forward selection [50] and another study (4%) with a stepwise algorithm [51]. In addition, there were mixed forms of variable selection used in some studies in which various methods were combined. Two studies (8%) first applied univariable screening and applied stepwise regression to the univariably significant variables; one of the studies used forward selection [52], the other backward elimination [53]. In two other studies (8%), some variables were introduced with background knowledge and some were included in the final model based on the univariable significance [54,55]. One study (4%) included variables based on background knowledge and additionally applied the shrinkage method LASSO to the selected variables [56]. Table 3 summarizes the used techniques for variable selection. One study (4%) further selected two instrumental variables [38].

Two studies (8%) considered possible non-linear functional forms of continuous independent variables. In one of the studies, they used splines to model the associations between CR use and the independent variables of age, body mass index, and procedure date [45]. The other study reported that generalized non-linear models were fitted [3]. Continuous variables were dichotomized in twelve studies (50%) [3,34,36,37,38,39,45,48,49,52,55,56]. Among others, the following variables were dichotomized: ejection fraction [3,34,39,45,49], age [38,52,55,56] and BMI [39,48,52].

In total, 13 studies (54%) used the propensity score (PS) method to adjust for confounders. Of these, ten studies (77%) calculated the PS using logistic regression [3,34,38,39,41,43,45,46,48,55] and three (23%) did not mention how the PS was calculated [44,53,56]. The propensity score was used for matching, stratification and in multivariable analysis (Table 4). Some studies (*n* = 5, 38%) used more than one of these methods and compared them. Seven studies (54%) included the PS in the regression model to adjust for potential confounders. Out of these, the PS was included as a covariate in the regression model in five studies (38%) [3,39,45,48,55], as the basis of inverse probability weighting in one study (8%) [56] and one study (8%) did not mention how the PS was included [46]. Furthermore, seven studies (54%) used the PS to perform matching [38,41,43,44,45,48,53] and four studies (31%) used the PS in a stratified analysis by subdividing the patients into five groups defined by quintiles of the PS [34,45,48,55].

The variable selection procedures for the PS were similar to the methods for the regression models. The selection methods are also summarized in Table 3.

The same study that applied spline methods added the PS into the multivariable model with a “3-df spline” [45]. Apart from that, no other study mentioned the linearity assumption regarding the estimation or usage of the PS.

One study mentioned multicollinearity [56]. Pair-wise interactions were mentioned in eight studies (33%) [38,41,45,48,49,51,55,56], although in two studies, it was unclear whether pair-wise interactions were included in the final models [45,55]. Ten studies (42%) addressed the problem of missing values [39,41,45,46,48,49,50,51,54,56] and reported the handling of missing values with varying degrees of detail. Four studies performed imputations of missing values. Of these, one study employed multiple imputation, but it was not described how, one study imputed with sample medians, another study imputed data using the Markov Chain Monte Carlo algorithm [46], and the last study applied multiple imputation by chained equation [49]. Mixed models were mentioned and used in two studies (8%). One of them included a random effects term in the model, unique for each matched pair of a CR and a non-CR patient [45], and the other study only mentioned that the NLMIXED procedure of SAS was used [3]. Based on the reported details, we were able to calculate the events per variable ratio (EPV) for only 16 studies (67%). We considered the number of all candidate variables as the denominator of EPV. In eight studies (33%) [3,36,37,46,47,51,52,55], an EPV lower than 15 was calculated, and in the other eight studies (33%) [39,40,41,45,48,49,54,56] we calculated an EPV of ≥15. Table 5 summarizes the general aspects that were screened within this review.

Overall, 18 (75%) studies built multivariable models [3,34,35,36,37,38,39,40,41,42,44,46,48,50,51,52,54,55,56], while six studies (25%) estimated both univariable and multivariable models [43,45,47,49,53]. In total, eleven studies (61%) used logistic regression with the intervention as outcome [3,34,35,39,41,45,46,48,50,55]. One study (8%) reported having applied a Cox-proportional hazards regression [35]. Twenty-two studies (92%) conducted a regression for the primary outcome (all-cause mortality), and of these, 16 (67%) applied Cox-proportional hazards regression [34,35,41,43,44,45,46,47,48,49,51,52,53,54,55,56] and six (25%) logistic regression [3,36,37,38,39,40].

For the twenty-two studies, we summarized the confounders that were used to either match the groups, derive a PS or were included in the final regression model (Supplementary S3 File). Age was selected in all studies, followed by gender, which was selected in all but two studies. Other confounders selected in at least half of the twenty-two studies were diabetes mellitus, hypertension, (prior) percutaneous coronary intervention, (prior) myocardial infarction/acute myocardial infarction, (prior) coronary artery bypass graft, ejection fraction, renal function/disease, peripheral vascular/artery disease and (congestive) heart failure.

Table 6 summarizes aspects of causal model building and initial data analysis, increasing the risk of bias if not properly conducted. Only studies that adjusted for confounders were included (*n* = 24). None of the studies used a DAG to select confounders. In one study (4%), the principle of including only pretreatment covariates was violated; for four studies (17%), this was uncertain. The common assumptions for deriving causal effects from observational studies, i.e., conditional exchangeability and positivity, were not mentioned in any study.

## 4. Discussion

This systematic methods review identified the standards of model building in the field of cardiac rehabilitation based on 28 studies published between 2004 and 2018. Our results are in line with those of previous systematic reviews on statistical methodology in other fields of medical research [57,58,59], demonstrating the widespread use of poor statistical modeling methods and incomplete reporting. This is despite the fact that CROS-II required that studies include a description of data sources and the use of methods to reduce risk and selection bias. Therefore, these studies were even pre-selected, methodologically “better” studies. The risk of bias assessment in CROS-II ([14], Table 3) already uncovered uncertainties in study protocols and issues related to reporting, selection bias, and confounder selection criteria in the evaluated CR cohort studies. In this article, we complemented their evaluation by assessing various aspects of statistical model building (Table 6). Adding a statistical risk of bias assessment increases the number of studies with a high risk of bias considerably.

Issues such as the handling of missing values, highly influential points or multicollinearity were also mentioned only partially or not at all in the screened studies. Highly criticized and not recommended methods were still applied; for instance, continuous variables were dichotomized in 50% of the studies.

One of the major issues is that many of the reviewed studies provided only limited information about model building. For example, in 11 out of 24 studies, it was not explicitly described how the set of independent variables had been derived. When it comes to data-driven variable selection, many authors have repeatedly pointed out the importance of using background knowledge, especially in a causal setting [8]. The adjustment set should ideally be established prior to patient recruitment to ensure that all confounders can be collected. Background knowledge was included in only one fifth of the studies. In any case, background knowledge should be used instead of data-driven methods for the identification of confounders.

For data-driven variable selection, a popular approach was the univariable selection method. Here, the association of each of the covariates potentially relevant for a model with the outcome is evaluated, while ignoring any other variables. Only those that are considered statistically significant are included in the multivariable model [60]. This selection procedure has the advantage of being very simple, which is probably why it is frequently used. However, it causes several problems as stated in Table 2. This univariable approach was already identified as problematic in 1996 [61], and yet 33% (*n* = 8) of the studies screened used this method to select variables. Stepwise procedures were also popular among the data-driven methods and were used in about 17% (*n* = 4) of the studies. They iteratively include or exclude covariates, one at a time, by assessing its significance at a pre-specified inclusion or exclusion level [8]. Forward selection starts with an empty, likely misspecified model, which is why it should be avoided. Backward elimination begins with a fully specified model and was the least frequently used of the stepwise procedures among the studies, despite being preferable to forward selection [62]. Methods for variable selection that have been criticized in the literature as problematic still prevail in the screened studies, background knowledge is barely used and no study used a DAG. Important concepts from the causal framework such as conditional exchangeability, positivity and consistency were not mentioned in a single study. These are assumptions that must be fulfilled in order to draw causal inferences [7,22]. Conditional exchangeability cannot be tested, and clinicians need to use their expert knowledge to enhance the credibility that the assumption is met. This underlines the importance of background knowledge for variable selection when considering causal aims. Furthermore, it is especially important to specify the functional forms of continuous variables in the model, which best reflect the true functional relationship between explanatory variables and outcome. In most medical applications, a simple linear relationship between continuous explanatory variables and response is assumed, which is often implausible [24,63]. For this purpose, various methods to estimate non-linear functional forms were introduced, including fractional polynomials and splines among others [64]. For Cox regression models, specific suggestions were also made [65]. Unfortunately, our study showed these still find very little to no application.

PS are an increasingly popular method to control for confounding in observational studies as the screened studies also showed. They are an alternative method to regression models and the same aspects of statistical model building for causal models as summarized in Table 2 need to be considered. While relatively little was reported on the previously mentioned aspects, there was a lot of information on the PS, addressing both calculation and adjustment method. The PS was most often calculated using logistic regression. However, as mentioned in the previous paragraph, if the relationship between the continuous covariates and treatment assignment is not linear, an appropriate nonlinear functional form must be considered in the propensity model. If not, the resulting PS may not be a good estimate of the true values and fail to achieve a good balance of the independent variables [66]. Regarding the specification of non-linear functional forms for the calculation of the PS, unfortunately only one study mentioned that splines were used, while the others did not report anything. Even in this particular study, spline regression was not clearly described, making it difficult to follow what was conducted.

Finally, it is important that the statistical methodology is clearly reported, in particular to assure replicability. If space limitations do not allow for sufficient details to be given in the methods section, an appendix or supplementary materials could describe the detailed statistical methodology. Certainly, the results are what readers are most interested in at the end, which may be the reason why the methods section often lacks precision. However, an intransparent or incomplete reported statistical methodology does not support the full replicability of the findings of a study ([67]; p. 99 f.) and often does not allow for the correct interpretation of estimated associations. Therefore, adequate reporting is essential.

Our goal to derive a robust adjustment model to assess the effect of the intervention cardiac rehabilitation on all-cause mortality could only be reached in parts, as the methods used for statistical model building were not sufficiently described and do not meet the state-of-the-art criteria in most studies. Furthermore, the exact aim of the studies was often not clear, as some referred to the covariates as predictors of mortality, which would then correspond to a prediction model. The terminologies are often misused and findings of different methods are conflated [68], leaving it unclear what the exact goal of the models is and whether they are really suitable for obtaining pooled estimates, as conducted in CROS-II.

## 5. Conclusions

The vast majority of the studies reviewed here do not meet the requirements for appropriate statistical model building as summarized in Table 2. The development of multivariable regression models is complex and needs interdisciplinary teams of researchers understanding the statistical methodology, being able to apply them appropriately and report them sufficiently. We can conclude that the methods used to develop models are in the majority of screened studies, not according to the state of the art and that the reporting often lacks precision. Background knowledge should be used as a selection criterion for confounders for explanatory models. Finally, non-linear functional forms need to be studied and simple linear or piecewise constant functional relations cannot always be assumed. While study authors cannot be blamed for using methods of causal inference which only from today’s perspective seem inferior to current approaches, one must still point out the possible erroneous conclusions from such studies [69]. This highlights the necessity of the continuous education of practitioners in statistical methodology. Instead of introducing more and more methodology, useful tutorial articles and trustworthy online resources and workshops are probably needed to guide applied researchers. While initiatives such as STRATOS try to help researchers to keep up with recent methodological developments by developing guidance documents, other endeavors like STROBE (STrengthening the Reporting of OBservational studies in Epidemiology) (https://www.strobe-statement.org/, accessed on 9 January 2023) try to improve the reporting of observational studies. We trust that this review will be a further step to improve the quality of the statistical methods used in causal research.

## Figures and Tables

**Table 1:** Summary of the main components of typical cardiac rehabilitation programs. CR programs usually contain subsets of these components.

Component	
Physical exercise	Supervised and structured exercise training at least twice a week [2]
Information	Advice on cardiovascular risk reduction [1]
Motivational techniques	Strategies to ensure patients adequately adhere to medications and implement lifestyle changes [3]
Education	Health education includes information on medication, exercise training, individual nutritional advice and support to stop smoking [3]
Psychological support and interventions	Psychological counselling, support of individual’s disease management and coping strategies, relaxation methods and individual behavior changes [3]
Social and vocational support	Support in social reintegration and reuptake of work [3]

**Table 2:** Criteria used to evaluate quality of statistical analysis and modeling of the studies.

*Quality Aspect*	Literature on Guidance	Assessment Procedure
**Selection of variables …**		
*…based on domain expertise and preceding studies (background knowledge)*	[21,22]	- reported in the methods section and performed appropriately (yes/no)
*…based on background knowledge with causal diagrams known as directed acyclic graphs (DAGs)*	[7,8,22]	- reported in the methods section and performed appropriately (yes/no)
**Assumption of …**		
*…pretreatment covariates.*	[7,22]	- reported in the methods section and performed appropriately (yes/no)
*…conditional exchangeability. This means that conditional on the IVs, association is causation.*	[7,22]	- reported in the methods section and discussed (yes/no)
*…positivity. This means each value of the confounders is observed for all treatment groups.*	[7,22,23]	- reported in the methods section and tested (yes/no)
*…consistency. This means that the mode of receiving, as opposed to the choice of treatment level per se, does not affect the outcome.*	[7,22,23]	- reported in the methods section and tested (yes/no)
*…linearity. This means that the relationship between a continuous IV and the outcome is linear.*	[24]	- reported in the methods section, possible non-linear relation investigated (yes/no)
*Missing values*	[20,25]	- proportion missing reported? (yes/no) - if necessary, appropriate analysis method applied (yes/no)
*Highly influential points/outliers*	[26]	- reported in the methods section - if necessary, appropriate analysis method applied (yes/no)
*Events-per-variable (EPV)*	[13], p. 72; [27,28]	- reported or possibility to calculate, sufficient EPV (>15) (yes/no)
*Dichotomization of continuous variables*	[29]	- appropriate analysis method applied (no dichotomization) (yes/yes, but not included in model/no)

**Table 3:** Variable selection methods used in 24 screened studies, of which 13 used a propensity score.

Selection Method *	for Regression (*n* = 24)		for Propensity Score (*n* = 13)	
	*n*	%	*n*	%
Background knowledge	5	21	2	15
Univariable screening	8	33	3	23
Stepwise selection	4	17	0	0
Shrinkage methods	1	4	1	8
Not described	11	46	8	62

* multiple items possible.

**Table 4:** Propensity score methods used in screened studies.

Propensity Score Methods	Studies (*n* = 13)	
	*n*	%
Matching	7	54
Stratification	4	31
Multivariable analysis included:		
as covariate	5	38
inverse probability weighting	1	8
inclusion not described	1	8

**Table 5:** General aspects of model building and reporting in screened studies.

	Studies (*n* = 24)	
	*n*	%
Missing values reported	10	42
Missing value imputation applied	4	17
Highly influential points reported	0	0
Multicollinearity reported	1	4
Pair-wise interactions reported	8	33
Pair-wise interactions applied	6	25
Mixed Models applied	2	8
Reconstruction of EPV possible	16	67
Dichotomization of continuous variables applied	12	50

**Table 6:** Risk of bias assessment based on the CROS-II quality assessment table extended by model-building aspects.

Study	Rauch 2014 [3]	Norris 2004 [34]	Kutner 2006 [35]	Milani 2007 [36]	Hansen 2009 [37]	Suaya 2009 [38]	Jünger 2010 [39]	Schwaab 2011 [40]	Martin 2012 [41]	Prince 2014 [42]	Doimo 2018 [43]	Sunamura 2018 [44]	Goel 2011 [45]	Marzolini 2013 [46]	Schlitt 2015 [47]	Pack 2013 [48]	Meurs 2015 [49]	Nielsen 2008 [50]	Alter 2009 [51]	Coll-Fernandez 2013 [52]	Lee 2016 [53]	Beauchamp 2013 [54]	Goel 2013 [55]	De Vries 2015 [56]
Confounders selected based on background knowledge?	NC	NC	NC	NC	NC	NC	N	NC	NC	NC	NC	NC	N	N	N	Y	Y	N	N	N	N	Y	Y	Y
Was a DAG used?	N	N	N	N	N	N	N	N	N	N	N	N	N	N	N	N	N	N	N	N	N	N	N	N
Only pretreatment covariates included?	Y	Y	NC	Y	N	Y	Y	Y	NC	NC	Y	Y	Y	Y	Y	Y	Y	NC	Y	Y	NC	Y	Y	Y
Exchangeability mentioned?	N	N	N	N	N	N	N	N	N	N	N	N	N	N	N	N	N	N	N	N	N	N	N	N
Positivity mentioned?	N	N	N	N	N	N	N	N	N	N	N	N	N	N	N	N	N	N	N	N	N	N	N	N
Consistency reported?	N	N	N	N	N	N	N	N	N	N	N	N	N	N	N	N	N	N	N	N	N	N	N	N
Incorporation of non-linear functional forms?	Y	N	N	N	N	N	N	N	N	N	N	N	Y	N	N	N	N	N	N	N	N	N	N	N
Missing data reported?	NC	NC	NC	NC	NC	NC	Y	NC	Y	NC	NC	NC	Y	Y	NC	Y	Y	Y	Y	NC	NC	Y	NC	Y
Missing data imputation?	NC	NC	NC	NC	NC	NC	Y	NC	NN	NC	NC	NC	Y	Y	NC	NC	Y	NN	NC	NC	NC	NN	NC	NC
Highly influential points/outliers?	NC	NC	NC	NC	NC	NC	NC	NC	NC	NC	NC	NC	NC	NC	NC	NC	NC	NC	NC	NC	NC	NC	NC	NC
Multicollinearity?	NC	NC	NC	NC	NC	NC	NC	NC	NC	NC	NC	NC	NC	NC	NC	NC	NC	NC	NC	NC	NC	NC	NC	N
Events-per-variable (EPV) >15	N	NC	NC	N	N	NC	Y	Y	Y	NC	NC	NC	Y	N	N	Y	Y	NC	N	N	NC	Y	N	Y
Dichotomization of continuous variables?	Y	Y	N	N	Y	Y	Y	NI	N	N	N	N	Y	NI	N	Y	Y	Y	N	Y	N	N	Y	Y

Y: yes; N: no; NC: not clear, not reported; NN: no need; NI: Yes, but not included in final model; green → adjudication is in favor of reliability of results and reporting; yellow → item potentially increases risk of limited reliability of results and reporting; red → item increases risk of reliability of results and reporting.

## Data Availability

Data can be requested from the first author.

## Supplementary Materials

The following supporting information can be downloaded at: https://www.mdpi.com/article/10.3390/ijerph20043182/s1, S1 Checklist. PRISMA reporting guideline. S1 File. Content sheet with summarized study and modeling characteristics. S2 File. Study characteristics. S3 File. Confounders summarized. References [2,14] are cited in the supplementary materials.
