# Supplementary material for: Causal Model Building in the Context of Cardiac Rehabilitation: A Systematic Review
Source: Int J Environ Res Public Health. 2023 Feb 11;20(4):3182. doi: 10.3390/ijerph20043182 (PMC9968189; doi:10.3390/ijerph20043182)
Supplement: Supplementary file 1 [file ijerph-20-03182-s001.zip › S1 File.pdf]

Aspects of Regression Modeling

Content Extraction Sheet for Article Screening

Name of reviewer:

Title of study	
Author of study	
Publication year of study	

	Level*
**1. Type of regression model**	
1.1 Univariable regression	*yes/no*
1.2 Multivariable regression	*yes/no*
1.3 Linear regression	*yes/no*
1.4 Logistic regression	*yes/no*
1.5 Cox regression	*yes/no*
1.6 Other	*free text*
**2. Propensity Score (PS)**	
2.1 PS estimation with logistic regression	*yes/no*
2.2 PS estimation with discriminant analysis	*yes/no*
2.3 Other estimation method	*free text*
2.4 Matching	*yes/no*
2.5 Stratification	*yes/no*
2.6 Covariate adjustment	*yes/no*
2.7 Inverse probability of treatment weighting	*yes/no*
2.8 Other usage	*free text*
**3. General aspects of regression modeling**	
3.1 Multicollinearity checked	*yes/no*
3.2 Interaction terms checked	*yes/no*
3.3 Interaction terms included	*yes/no*
3.4 Missing values checked	*yes/no*
3.5 Missing value imputation	*yes/no*
3.6 Imputation method	*free text*
3.7 Mixed models	*yes/no*
3.8 Highly influential points	*yes/no*
3.9 Number of explanatory variables in full model	*number*
3.10 Number of explanatory variables in full model with level of categories (degrees of freedom)	*number*
3.11 Number of events	*number*
3.12 EPV	*number*
3.13 Only pretreatment covariates included	*yes/no*
3.14 Conditional exchangeability discussed	*yes/no*
3.15 Positivity checked	*yes/no*
3.16 Consistency discussed	*yes/no*
**4. Selection of variables**	

4.1 Adjustments but no information on variable selection	*yes/no*
4.2 Based on background knowledge	*yes/no*
4.3 Univariable	*yes/no*
4.4 Stepwise procedure	*free text*
4.5 Shrinkage method	*free text*
4.6 Other	*free text*
**5. Functional form of continuous predictors**	
5.1 Possibility of a nonlinear relation	*yes/no*
5.2 Non-linear functional forms included	*yes/no*
5.3 If yes, which method	*free text*
5.4 Dichotomization of continuous predictors	*yes/yes, but not included in model/no*

*If information is not available, the aspect is left empty
