# Supplementary material for: Causal Model Building in the Context of Cardiac Rehabilitation: A Systematic Review
Source: Int J Environ Res Public Health. 2023 Feb 11;20(4):3182. doi: 10.3390/ijerph20043182 (PMC9968189; doi:10.3390/ijerph20043182)
Supplement: Supplementary file 1 [file ijerph-20-03182-s001.zip › S2 File.pdf]

Study Characteristics summarized

ID	Author	Year	Title	Study Design	retro-/ prospective (r/p)	Total Sample Size
1_1	Boulay	2004	Health-care consumption and recurrent myocardial infarction after 1 year of conventional treatment versus short- and long-term cardiac rehabilitation	OS	r/p	128
1_2	Norris	2004	Referral Rate and Outcomes of Cardiac Rehabilitation After Cardiac Catheterization in a Large Canadian City	OS	r	5081
1_3	Kutner	2006	Cardiac Rehabilitation and Survival of Dialysis Patients after Coronary Bypass	OS	r	6215
1_4	Milani	2007	Impact of Cardiac Rehabilitation on Depression and is Associated Mortality	OS	r	701
1_5	Nielsen	2008	Cardiac rehabilitation: health characteristics and socio-economic status among those who do not attend	OS	r	200
1_6	Alter	2009	Relationship between cardiac rehabilitation and survival after acute cardiac hospitalization within a universal health care system	OS	r	4084
1_7	Hansen	2009	Reduction of cardiovascular event rate: different effects of cardiac rehabilitation in CABG and PCI patients	OS	p	677
1_8	Suaya	2009	Cardiac Rehabilitation and Survival in Older Coronary Patients	OS	r	601099
1_9	Jünger	2010	Effect of early short-term cardiac rehabilitation after acute ST-elevation and non-ST-elevation myocardial infarction on 1-year mortality	OS	r	4547
1_10	Goel	2011	Impact of Cardiac Rehabilitation on Mortality and Cardiovascular Events After Percutaneous Coronary Intervention in the Community	OS	r	2395
1_11	Kim	2011	Prognostic Influences of Cardiac Rehabilitation in Korean Acute Myocardial Infarction Patients	OS	p	141
1_12	Schwaab	2011	In-patient cardiac rehabilitation versus medical care – a	OS	r	1474

			prospective multicentre controlled 12 months follow-up in patients with coronary heart disease			
1_13	Martin	2012	Cardiac rehabilitation attendance and outcomes in coronary artery disease patients.	OS	p	5886
1_14	Beauchamp	2013	Attendance at cardiac rehabilitation is associated with lower all-cause mortality after 14 years of follow-up	OS	r	544
1_15	Lee	2013	Regular exercise training reduces coronary restenosis after percutaneous coronary intervention in patients with acute myocardial infarction	OS	p	74
1_16	Marzolini	2013	Outcomes associated with cardiac rehabilitation participation in patients with musculoskeletal comorbidities	OS	p	851
1_17	Pack	2013	Participation in Cardiac Rehabilitation and Survival After Coronary Artery Bypass Graft Surgery	OS	r	846
1_18	Coll-Fernandez	2014	Cardiac Rehabilitation and Outcome in Stable Outpatients With Recent Myocardial Infarction	OS	p	1043
1_19	Prince	2014	Racial Disparities in Cardiac Rehabilitation Initiation and the Effect on Survival	OS	r	822
1_20	Rauch	2014	Short-term comprehensive cardiac rehabilitation after AMI is associated with reduced 1-year mortality: results from the OMEGA study	OS	p	3560
1_21	Goel	2013	Cardiac rehabilitation is associated with reduced long-term mortality in patientsundergoing combined heart valve and CABG surgery	OS	r	201
1_22	De Vries	2015	Cardiac rehabilitation and survival in a large representative community cohort of Dutch patients	OS	r	35919
1_23	Meurs	2015	The association between cardiac rehabilitation and mortality risk for myocardial infarction patients with and without depressive symptoms	OS	r	1702

1_24	Schlitt	2015	Anschlussheilbehandlung bei Patienten mit koronarer Herzkrankheit	OS	r	1910
1_25	Lee	2016	Impact of exercise-based cardiac rehabilitation on long-term clinical outcomes in patients with left main coronary artery stenosis	OS	p	3120
1_26	Espinosa Caliani	2004	Rehabilitación cardíaca postinfarto de miocardio en enfermos de bajo riesgo. Resultados de un programa de coordinación entre cardiología y atención primaria	OS	p	153
1_27	Doimo	2018	Impact of ambulatory cardiac rehabilitation on cardiovascular outcomes: a long-term follow-up study	OS	r	1280
1_28	Sunamura	2018	Cardiac rehabilitation in patients with acute coronary syndrome with primary percutaneous coronary intervention is associated with improved 10-year survival	OS	r	2318

OS=observational study
